# Demonstration of charge-hold-vent (CHV) and no-vent-fill (NVF) in a simulated propellent storage tank during tank-to-tank cryogen transfer in microgravity

**DOI:** 10.1038/s41526-024-00403-6

**Published:** 2024-06-06

**Authors:** J. N. Chung, Jun Dong, Hao Wang, Bo Han Huang, Jason Hartwig

**Affiliations:** 1 Cryogenics Heat Transfer Laboratory, Department of Mechanical and Aerospace Engineering, University of Florida, Gainesville, FL 32611-6300 USA; 2 NASA Glenn, Research Center, Cleveland, OH USA

**Keywords:** Engineering, Environmental sciences

## Abstract

The space exploration from a low earth orbit to a high earth orbit, then to Moon, Mars, and possibly asteroids and moons of other planets is one of the biggest challenges for scientists and engineers for the new millennium. The enabling of in-space cryogenic rocket engines and the Lower-Earth-Orbit (LEO) cryogenic fuel depots for these future manned and robotic space exploration missions begins with the technology development of advanced cryogenic thermal-fluid management systems for the propellant transfer line and storage tank system. One of the key thermal-fluid management operations is the chilldown and filling of the propellant storage tank in space. As a result, highly energy efficient, breakthrough concepts for quenching heat transfer to conserve and minimize the cryogen consumption during chilldown have become the focus of engineering research and development, especially for the deep-space mission to Mars. In this paper, we introduce such thermal transport concepts and demonstrate their feasibilities in space for cryogenic propellant storage tank chilldown and filling in a simulated space microgravity condition on board an aircraft flying a parabolic trajectory. In order to maximize the storage tank chilldown thermal efficiency for the least amount of required cryogen consumption, the breakthrough quenching heat transfer concepts developed include the combination of charge-hold-vent (CHV) and no-vent-hold (NVF). The completed flight experiments successfully demonstrated the feasibility of the concepts and discovered that spray cooling combined with hold and vent is more efficient than the pure spray cooling for storage tank chilldown in microgravity. In microgravity, the data shows that the CHV thermal efficiency can reach 39.5%. The CHV efficiency in microgravity is 6.9% lower than that in terrestrial gravity. We also found that pulsing the spray can increase CHV efficiency by 6.1% in microgravity.

## Introduction

The space exploration from a low earth orbit to a high earth orbit, then to Moon, Mars, and possibly asteroids and moons of other planets is one of the biggest challenges for scientists and engineers for the new millennium. An indispensable part to these missions is the effective, affordable, and reliable supply of cryogenic propellant fluids, and their thermal management in space. The efficient and safe utilization of cryogenic propellant fluids in thermal management, power and propulsion, and life support systems of a spacecraft during space missions involves the transport, handling, and storage of these fluids in terrestrial, reduced gravity and microgravity conditions.

Lower-earth-orbiting (LEO) propellant fuel depots planned for future deep-space missions and human-carrying orbital transfer spacecraft flying to the moon and Mars will have to utilize the high thrust and high efficiency of liquid cryogenic chemical propulsion or nuclear thermal propulsion^1–4^. Efficient in-space tank-to-tank propellant transfer (propellant fuel depot to orbital transfer spacecraft) of cryogenic fluids is an enabling technology for the deep-space missions. The transfer of cryogenic propellants in space, however, has yet to be accomplished, partially due to the unavailability of cryogenic quenching heat transfer data during chilldown (quenching) and filling of the propellant receiver tank in reduced gravity and microgravity^4^ as liquid propellant cannot be stored in a liquid state until the tank is quenched down to the liquid temperature. The consumed propellant during chilldown is a mixture of vapor and liquid that cannot be used for any useful purpose and therefore must be vented overboard. Since the current terrestrial cryogenic tank chilldown and filling technology can only manage to offer relatively very low thermal energy efficiencies^5^ and further that it has never been developed under space microgravity conditions, a new break-through technology advance that significantly raises the efficiencies for tank chilldown and filling, and is also verified under space conditions is needed for enabling deep space missions.

According to publications by the members of the Space Cryogenic Thermal Management Group at NASA Glenn Research Center, Doherty et al. ^6^ and Meyer et al. ^2^ provided the main areas of Research and Development for space cryogenic thermal management. In-space tank-to-tank transfer thermal management is composed of three operational elements – (1) high efficiency transfer line chilldown, (2) high efficiency tank chilldown, and (3) no-vent fill of the receiver tank. When transferring a cryogenic propellant from a supply tank to a receiver tank, the transfer line (i.e. pipe and valves) and receiver tank are at high temperatures compared to that of the liquid propellant. Chilldown is the process of introducing the cryogen into the system to cool the hardware down to the liquid propellant temperature.

Among all three areas, the receiver tank chilldown and fill are considered the most important area as the amount of cryogen consumed is the largest. These previous microgravity studies^7–12^ have focused solely on the transfer line chilldown of pipes. More difficult to model and in most cases more important for the propellant thermal management is the chilldown and fill of the receiver tank. The receiver tank is the most massive component of the system to chill down and, therefore, takes much more time to chill down and thus more cryogen consumption. As a result, the time and propellant spent during tank chilldown will mostly be dictated by how fast the tank can be chilled down. Previous tank chilldown studies have been limited to just 1 g conditions and were very scarce with only two data sets available and the experiments were poorly executed^13^. Only recently, the current authors’ group has published microgravity results only for spray cooling chilldown of a simulated propellant storage tank wall^14^ and a simulated storage tank^15^, respectively. However, the current paper addresses the specific procedure of Charge-Hold-Vent (CHV) and No-Vent-Fill (NVF) for tank-to-tank cryogenic transfer.

Like in the chilldown of a transfer line, the temperature of the receiver tank walls must be quenched down to below the liquid saturation temperature to ensure that single-phase liquid can be maintained in the tank. A significant initial portion of the cryogen is quickly vaporized from absorbing the thermal energy released from the tank walls. To avoid over-pressurization, on earth the vent valve can be left open so that the vapor is vented into the outside environment. But, in microgravity this poses a problem since inadvertent venting of lingering liquid is possible, as the liquid is expected to adhere to the tank walls or to float around. Only two sets of ground tests have been carried out that show the feasibility of a charge-hold-vent (CHV) procedure followed by a no-vent fill (NVF) procedure^16,17^. CHV consists of charging a small amount of liquid into the tank to chill down the tank wall, holding the vent valve closed to allow the liquid to completely vaporize, and then opening the vent valve to relieve the pressure. This three-step procedure is repeated until the tank wall temperature is sufficiently chilled down below the liquid saturation temperature. After this point, NVF is performed which consists of closing the vent valve and continuously loading the tank with liquid propellant. Using these two methods together, an efficient and safe low-gravity tank-to-tank transfer is possible.

The challenge in CHV is the ability to predict the two-phase heat transfer during the quenching of tank wall. Knowledge of the quenching heat transfer on the tank wall will help determine the length of time it takes for the liquid charge to cool the wall and vaporize. This will help in the design of the flight software control logic to carry out the tank-to-tank transfer. Time can also be a critical factor in some mission scenarios that rely on strict scheduling of mission events. Thus, leaving a large uncertainty in the heat transfer rates can put an unnecessary strain on mission design. Accurate predictions of the heat transfer rates will also enable the design of optimal quenching methods (e.g. spray or jet impingement cooling) and flow conditions (e.g. flow rate, subcooling) for minimizing propellant waste and chilldown time.

Progressive advances in high power density electronics, and high-performance energy systems have precipitated the need for innovative thermal management technologies to ensure reliable performance and reduce the payload of thermal management systems. Such systems include high current density propulsion systems, high power electronics for energy conversion, high power optical sensors, as well as high power microelectronics packaged within environmental enclosures. In order to manage the progressively increasing heat flux requirements for thermal management systems, spray cooling system has been proposed and under constant development for the past sixty years. Based on the above, spray cooling was chosen as the chief heat transfer method for quenching the tank wall during CHV in the current study. As mentioned above, microgravity spray cooling of tank wall has been rigorously researched by the current authors’ group^14,15^.

In CHV, at the beginning, the tank wall is quenched down by spray cooling or jet impingement that results in the pressurization of the tank by vapor generated. Current models have been developed and fitted to ground data with some success^13^. But it continues to be an area of developing research due to the complexity of the problem. Since it has been shown from simple boiling experiments and pipe chilldown tests in low gravity that the heat transfer in two-phase flows is heavily dependent on gravity level^7,18^, it is anticipated that the heat transfer characteristics in CHV will be significantly different in low gravity from that in 1-g as well. Therefore, it is imperative that low gravity data be obtained to help anchor microgravity CHV heat transfer models.

During NVF, the liquid first flashes off as the tank wall is still warmer than the saturation temperature. Once the tank pressure exceeds the saturation pressure of the liquid, the liquid begins to accumulate in the tank. As the pressure continues to increase, the vapor begins to condense into the liquid which allows more liquid to enter the tank. This process continues until the tank pressure reaches the maximum allowable pressure of the tank or until the critical pressure of the cryogen, whichever is lower. The complex behavior of the NVF process occurs during the vapor condensation period. The goal is to disturb the liquid-vapor interface in order to enhance the rate of condensation, and thus the rate at which the tank can be filled. Spray nozzles are envisioned as the one method to disturb the interface and increase the overall interfacial area. Their performance, however, is highly dependent on whether or not they are submerged under the liquid^13^. The behavior in 1 g is not directly applicable to low gravity, due to the lack of buoyancy force to stir the vapor. Also, in low gravity it is not easy to keep a spray nozzle from being submerged in liquid, since the liquid tends to stick to solid surfaces.

Based on the current state of the art on CHV and NVF, the following objectives were chosen for the current research:

1. Provide first-ever feasibility demonstration of cryogenic CHV and NVF for tank chilldown and fill in microgravity.
2. Provide a valuable set of microgravity tank chill and fill data over a range of experimental conditions for future model development and verification of numerical simulations.

## Methods

### Experimental system

**Fig. 1:**
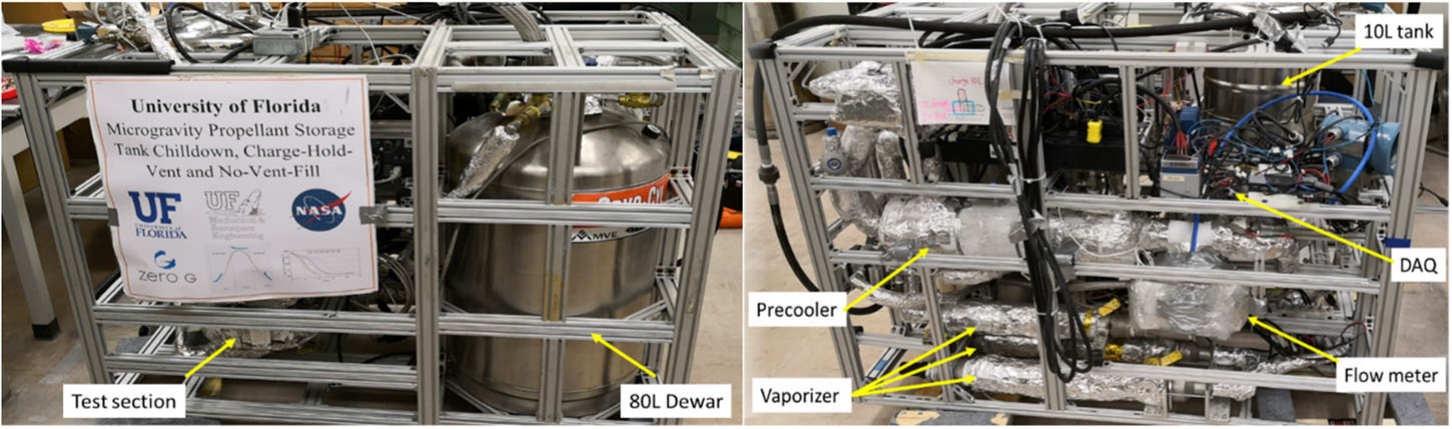
Photographic images of the experimental system. Left is front view and right is back view.

Figure 1 provides the photos of the experimental system. The system was used for both terrestrial experiment and microgravity parabolic flight experiment. All the system components except the high-pressure gas cylinder fit into a 1.4 m × 0.8 m × 1 m (L × W × H) 8020 aluminum frame. For the flight experiment, this highly integrated thermal-fluid system was installed on the floor of ZERO-G Corporation’s Boeing 727–200 F aircraft^19^ to perform the parabolic flight tank chilldown experiment in a simulated microgravity environment. The reduced gravity is achieved through flying the aircraft in a parabolic trajectory (Fig. 9) and each parabola provides about 17–20 s reduced gravity (10^−2^g) period.

**Fig. 2:**
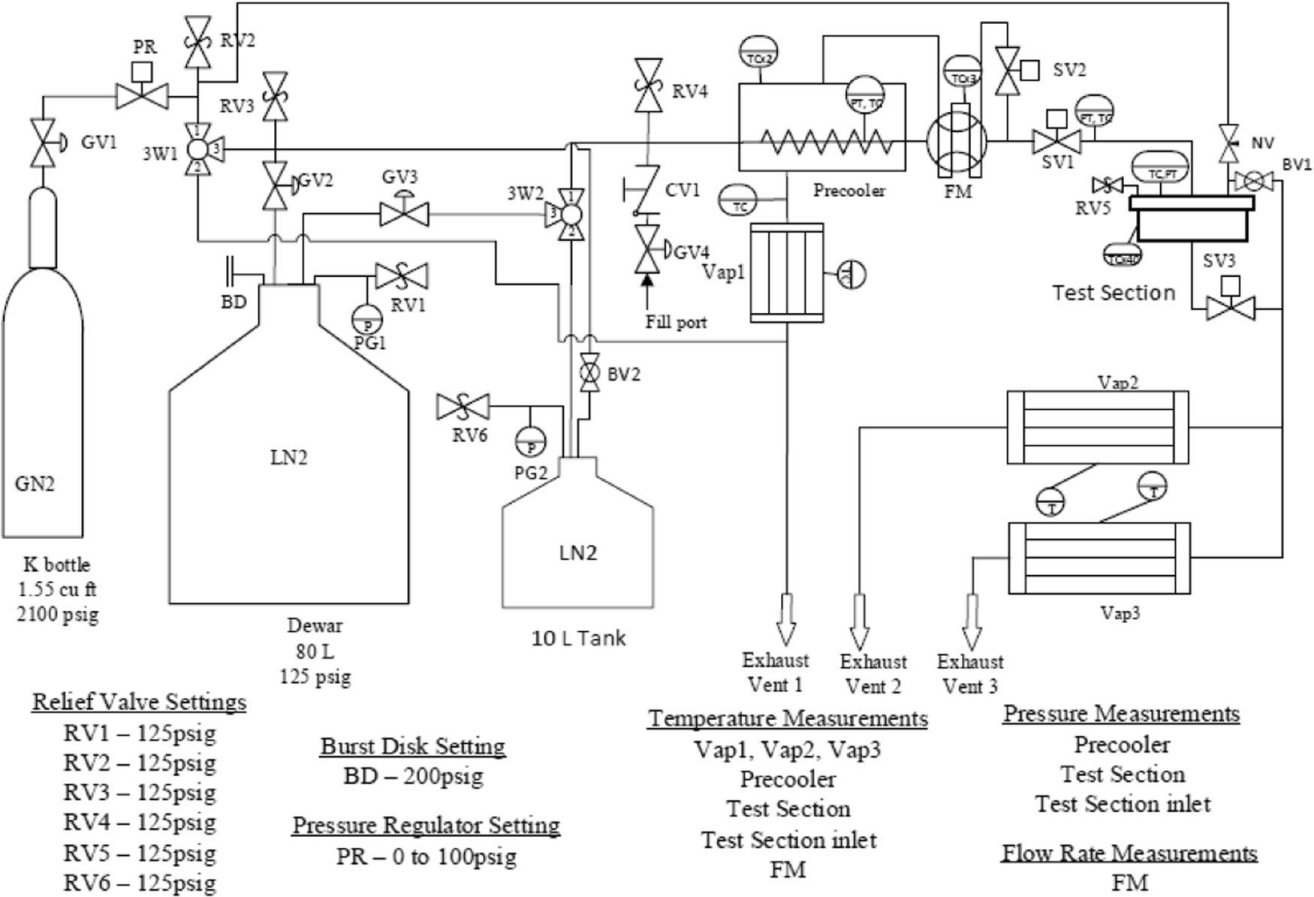
Fluid system schematic. The valves and important components of the fluid network. Relief valve settings, the burst disk setting, and pressure regulator settings are also included. BD burst disk, BV ball valve, CV check valve, FM flow meter, GN2 gaseous nitrogen, GV globe valve, LN2 liquid nitrogen, NV needle valve, PG pressure gauge, PR pressure regulator, PT pressure transducer, RV relief valve, SV solenoid valve, TC thermocouple, Vap vaporizer, 3V three-way valve.

**Fig. 3:**
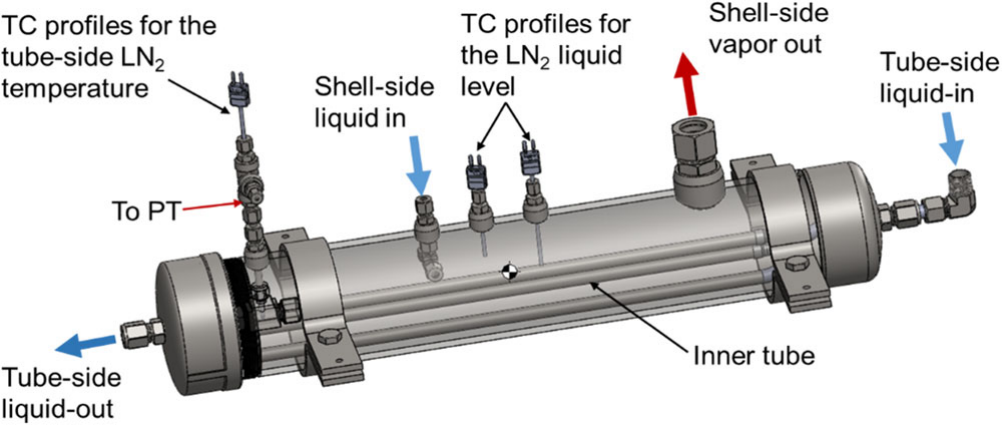
A CAD drawing of the subcooler. It is a shell and tube heat exchanger.

The experimental apparatus consists of four essential fluid units together with auxiliary components, fluid piping and instrumentations. The fluid piping schematic and instrumentation diagram is shown in Fig. 2. The 80-L double-walled cryogenic dewar supplies the LN2 to the subcooler (also called precooler) and the test section for performing the tank chilldown test as well as provides the LN2 for the pre-chilling of all the fluid components upstream of the test section prior to the actual tank chilldown test. Before the experiment, the 80 L dewar is topped off with industrial grade liquid nitrogen from a standard Airgas 180-Liter dewar through the LN2 fill port. The subcooler is essentially a simple shell-tube heat exchanger (Fig. 3) and it serves two functions. The first one is to subcool the liquid nitrogen before it enters the test section such that the thermodynamic state of the liquid entering the test section can be determined. During the subcooler operation, the inner tube of the subcooler is totally submerged in the liquid nitrogen bath on the shell-side. Since the pressure inside the tube that is measured by a pressure transducer is always higher than that on the shell side, the liquid nitrogen bath is always colder than that inside the tube. Thus, heat is removed from the liquid in the tube side. The second function is to conserve the liquid nitrogen during the pre-test chilldown of the upstream components of the test section. The vapor generated on the shell side is separated by gravity and is vented outside the system.

**Fig. 4:**
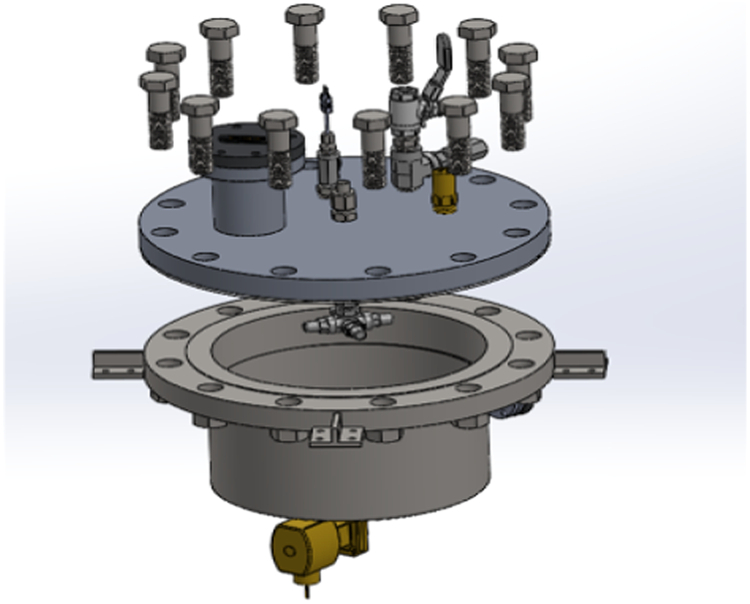
A CAD drawing of the test section. It is a chamber with spray nozzzles.

**Fig. 5:**
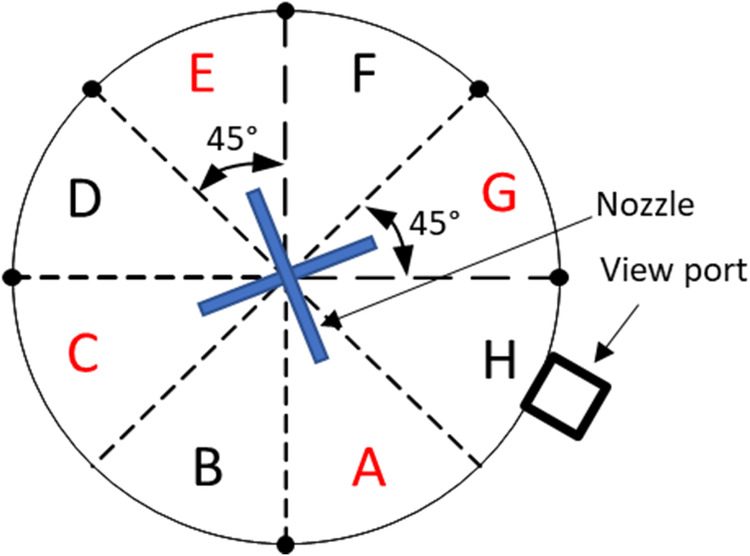
A schematic of the cross section of test chamber. It shows the layout of thermal couples.

**Fig. 6:**
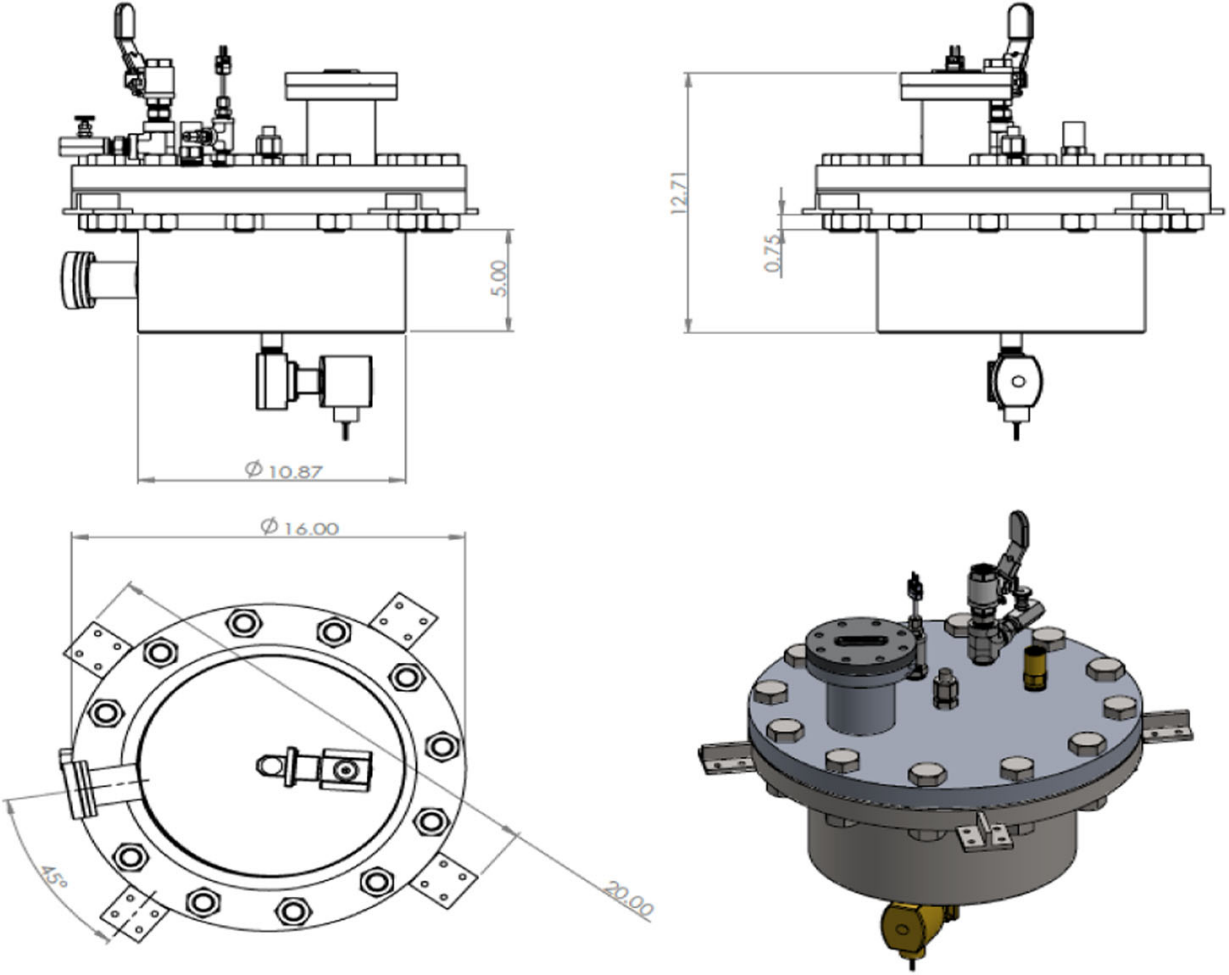
Test tank CAD drawings. They show the desgns with dimensions in inches (1 inch = 2.54 cm).

**Fig. 7:**
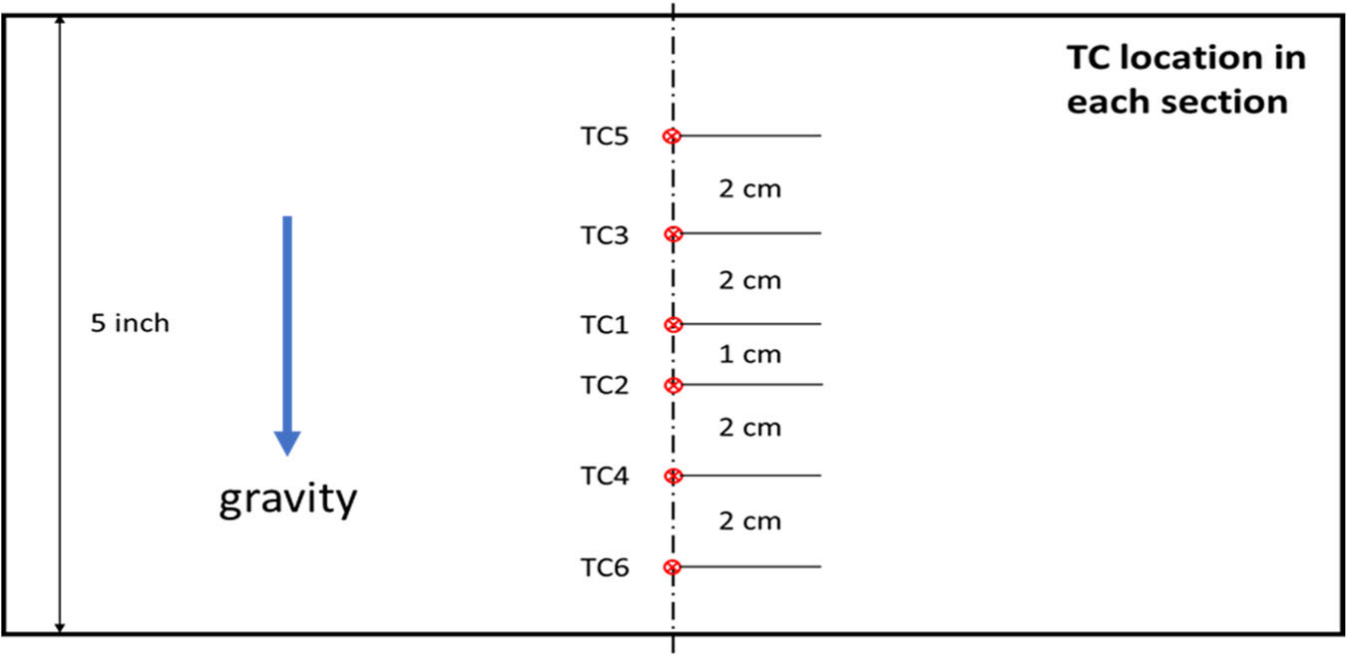
Thermal couple locations. The thermal couples are lined up in vertical direction on the outer surface of test chamber.

As shown in the CAD drawing of Fig. 4, the test tank of this apparatus is designed to comprise four nozzles to spray liquid nitrogen onto four separate tank wall sections (A, C, E, G shown in Fig. 5) simultaneously. The mass flow rates from the four nozzles are assumed identical as the four nozzles are identical and sharing a common source fluid supply. Additionally, the flow paths are symmetric. The test tank is insulated on the outside with aerogel layers. The test section is basically a cylindrical tank where the cryogenic spray cooling of the tank wall takes place during the experiment and it is built mostly with commercial off-the-shelf components. The exploded view of the test section is given in Fig. 4, which shows the configuration of the four spray nozzles. The tank design and dimensions are given in Fig. 6 where the length is in inches. These four spray nozzles are placed at the center inside the 10.75” (27.3 cm) inner diameter and 5” (12.7 cm) high cylinder tank (Fig. 5). For measuring the tank wall transient temperature history for each of the seven sections (A, B, C, D, E, F, and G as shown in Fig. 5) during the chilldown, a set of 6 thermal couples (TCs) are soldered on the outer surface of each tank wall section (backside). These 6 thermal couples and the view port in Section H are insulated from the ambient air by tank wall aerogel insulation layer. The 6 TCs are lined up vertically in the middle of each section as shown in Fig. 7. Specifically, TC 1 is directly lined up and pointed to the nozzle as shown in Fig. 7. Omega polyimide film heaters (KHA-404/5) are attached to the outside surface of the tank wall to reheat the stainless steel wall back to the initial temperature for the next test after each chilldown test.

The flow coming out of the test tank goes into two separate vaporizers in parallel (labeled as Vap2 and Vap3 in Fig. 2). The vaporizers are basically heat exchangers made from tube bundles, which evaporate any remaining liquid nitrogen in the two-phase flow coming out of the test section and also heat up the cold nitrogen vapor to above 0 °C before venting pure warm vapor out of the system. Each vaporizer is built by packing eight grooved copper tubes that have star shaped inner insertions on a 2.5” (6.35 cm) schedule 40 stainless steel pipe. The tube bundles are heated up to 200 °C before each test by a high temperature heating tap that is wrapped around the outer surface of the stainless steel pipe. The Labview program monitors and controls the “on and off” of the heating tap by the combination of K type thermocouples, NI 9211 thermocouple input module, NI 9472 digital output module, and mechanical relays. If the experiment is performed on board the aircraft, the gas coming out of the vaporizers is vented outside the aircraft cabin through rubber hoses that connect to the vent ports on the cabin wall. Similarly, another vaporizer, Vap1 ensures the proper venting of gaseous nitrogen coming out of the subcooler.

The data acquisition system including Labview program and National Instrument Compact DAQ hardware collects all sensor data and displays them real time on a laptop at a sampling rate of 16 Hz. NI 9214 TC modules read all the output from T-type TCs (Omega). NI 9205, an analog input module, reads all the voltage signals from pressure transducers (Omega PX 409V5A) and the 4–20 mA current signals (through a 249 ohm resistor) from the Coriolis liquid flow meter (Micro motion CMF025). The Labview program controls the opening and closing of the solenoidal valve, SV1, through a combination of NI USB 6009 and Solid-State relay. In the case of continuous spray, the relay energizes the solenoid valve after receiving a continuous voltage signal. However, in the case of intermittent spray, the relay energizes and de-energizes SV1 according to a rectangular waveform voltage signal from the Labview Virtue Instrument.

In the current experiment, the test tank wall is made of 0.06” (0.15 cm) thick stainless steel 304 sheet. The test tank was highly insulated on the outer surface with aerogel insulation. Additionally, the inner surface of the tank wall was coated with a 71-micron thick layer of low-thermal conductivity Teflon material. Specifically, the coating material was Fluorinated Ethylene Propylene (FEP) made by DuPont and classified by DuPont as Teflon 959G-203 that is a black color paint and has a thermal conductivity of 0.195 W/mK (DuPont publication^20^). The cryogenic tolerant coating was sprayed on to the inner side of the tank. The coating thickness was estimated at 70 microns by the coating company.

### Spray nozzle

**Fig. 8:**
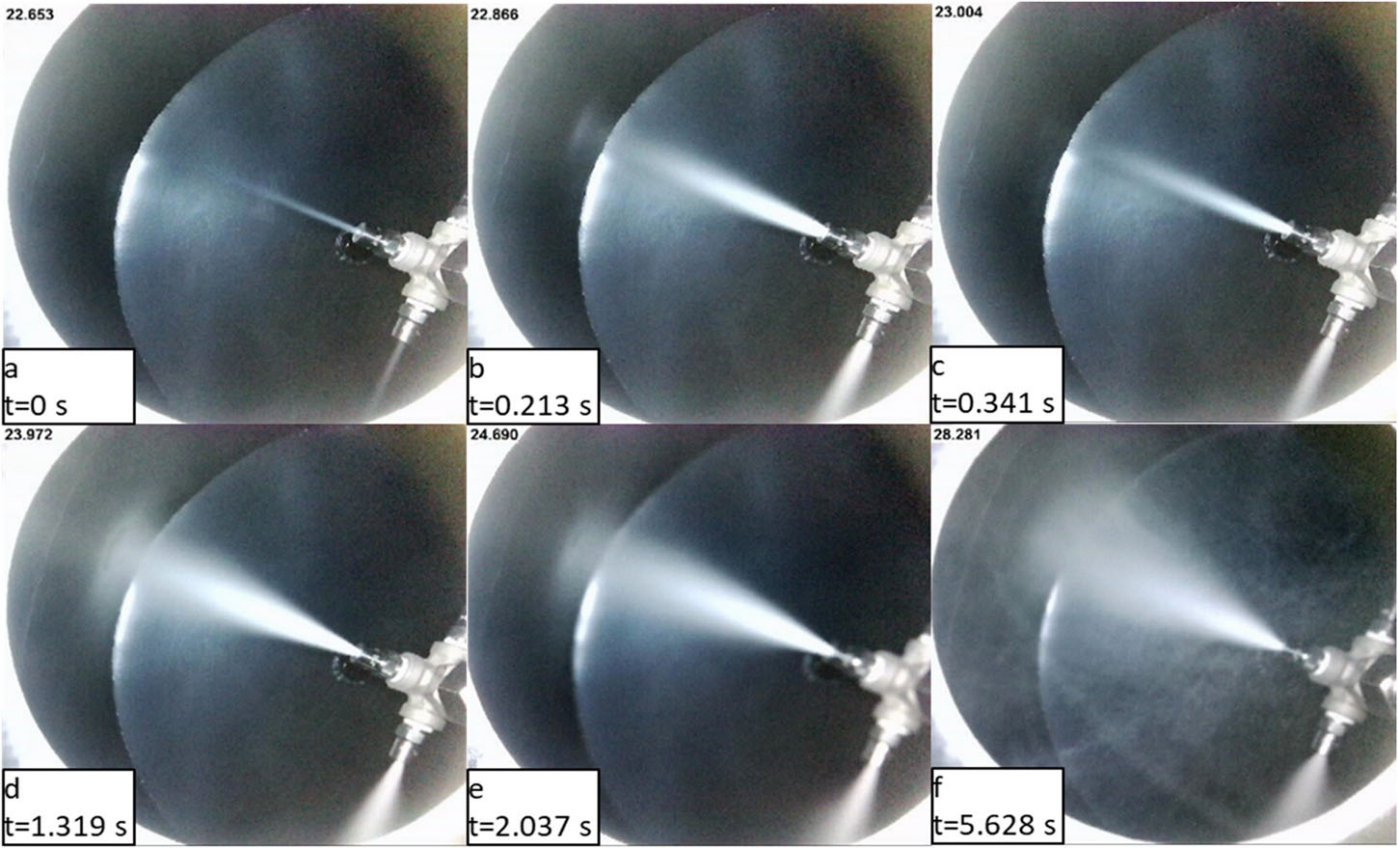
Flow visualization of cryogenic spray angle development. **a** t = 0 s, **b** t = 0.213 s, **c** t = 0341 s, **d** t = 1.319 s, **e** t = 2.037 s, **f** t = 5.628 s.

For the spray quenching of the tank surface, a full cone pressure-driven nozzle was chosen due to its simplicity and uniform coverage of the spray area. The Bete nozzle Model 1/8WL-3/4 90 was selected for our microgravity experiment. The connection tube size is 1/8 inch and the rated mass flow rate for water is 0.75 GPM under 40 psig injection pressure. The nozzle is made of 316 stainless steel. The rated spray cone angle is 90^o^ for water under 40 psig injection pressure. However, for our liquid nitrogen application, we found that spray cone angle is about 45^o^ for the fully developed spray with an injection pressure of 80 psig as shown in Fig. 8f.

In general, the spray angle is affected by the injection pressure and the characteristics of the spray nozzle. Therefore, test runs were performed first to determine the spray angle using liquid nitrogen as the working fluid. We found that the fully developed spray angle increases with the injection pressure. Figure 8 shows the visualization photographs of spray angle development using liquid nitrogen as the working fluid. Figure 8a is the frame taken right after the start of the spray. For Fig. 8a, b, c, they show the initial spray profile development. The further spray development is shown in Fig. 8d, e, f where the spray angles (cones) were expanding more before reaching the steady state. The spray was finally fully developed and stabilized at about t = 5.63 s. The fully developed spray angles are 45^o^ and 55^o^ under injection pressures of 80 psig (551 kPag) and 100 psig (689 kPag), respectively.

### Experimental procedure

For our parabolic flight experiment, the aircraft flies 5 parabolas in a row and takes a 3-minute break before the next 5-parabola set. The Zero-G corporation provides a total of 6 parabola sets (a total of 30 parabolas in a single flight). Under the constraint of the parabolic flights, we made sure that the key steps of CHV, i.e. Charge, Hold, and Vent were executed in microgravity. For each 80-second parabola, a 17-second microgravity period is embedded. For each microgravity period of CHV operation, it will begin with a 3-second charge of fluid in the form of spray, that is followed by a 17-second hold and then vent. It is these critical 17 s of microgravity periods during each parabolic trajectory cycle which allowed the experimenters to study the collective effect of microgravity on the ‘Charge (Spray Cooling)’ and ‘Hold’ phases of the CHV process and thereby quantify and compare the resulting chilldown efficiency achieved by the CHV in microgravity compared to 1 g environments. To run the system for the CHV, mainly four steps are followed, and these are initial system start-up, pre-cooling, CHV testing, and tank reconditioning by reheating. The initial start-up is the step where all the electrical devices are turned on. This includes running the pre-programed Labview script and turning on the vacuum pump (Turbo Lab 80). Once the Labview script is running, it will automatically set the output voltage of the DC power supply for the pressure transducers, and then turn on the heating cables of the vaporizers. This step takes about 30 min mainly due to the time required for heating up the vaporizers to 200 °C. Meanwhile, the globe valves, GV1, GV2, GV3 are open for the next step. The second step involves the pre-cooling and pre-chilling of all the piping and fluid components upstream of the test section to make sure that only liquid phase working fluid will enter the test section. The system proceeds first by rotating the three-way valve, 3V1, from GV1 to GV2, and setting the desired testing pressure on the pressure regulator, PR. Once the solenoidal valve SV2 is open by clicking the virtual button on the Laptop screen, then the liquid nitrogen starts to flow from the 80-L dewar into the shell-side of the subcooler. Before the liquid nitrogen can fill up the shell-side of the subcooler, the flow path upstream of the test section has to be chilled down completely. This step prevents the boil-off of liquid nitrogen before it flows into the test section. Once the inner tube of the subcooler is full (can be determined by the reading of the TC located inside the subcooler), the system is ready for the CHV experiment. The CHV test is started simply by clicking the virtual start button on the laptop screen, then the solenoid valve, SV1, will be opened according to the preset waveform signals either to continuously or intermittently supply nitrogen into the test section for spraying on the tank wall. After the pre-set period of the “charge” phase, the solenoid valve, SV1, is then closed by clicking the virtual stop bottom. At the end of the 5-parabola run, the reheating step starts to prepare the stainless steel tank wall for the next test cycle. To heat up the tank wall to the initial temperature (280K–300K), the film heaters are turned on by clicking the heating virtual bottom on the screen. Once the tank wall is heated back to the room temperature, the film heaters are turned off. This marks the readiness for the next cycle of testing which starts with the pre-cooling step.

**Fig. 9:**
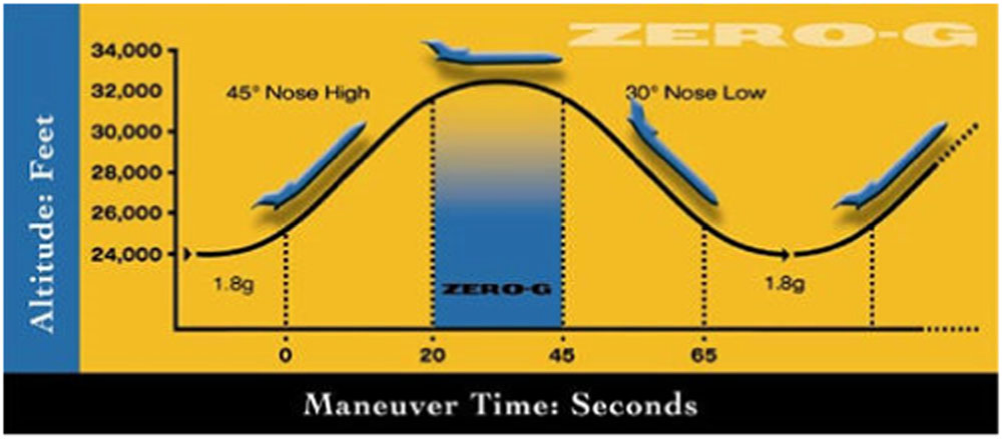
Schematic of parabolic flight trejectory. It shows the variable gravity levels vs. time.

In addition to the experimental procedure discussed above, next we need to mention the simulated microgravity environment on the parabolic flight. The variable gravity condition inside the airplane was created when the airplane is flying a parabolic trajectory. Figure 9 provides the information on the parabolic flight gravity-level versus time characteristics^19^. The microgravity period is always sandwiched by two 1.8-g periods where g is the earth gravity of 9.81 m/s^2^. The microgravity period nominally lasts between 18 and 25 s for commercial flights. For our research flights, in order to maintain the acceleration levels within ±0.01 g, the microgravity period is around 17–20 s.

### Experimental uncertainty

Table 1 lists all the uncertainties for the independently measured quantities. The uncertainty for the chilldown thermal efficiency is discussed in the results section.

**Table 1:** Measured quantities and uncertainties

Symbol	Quantity	Measurement method	Uncertainty
T	Tank wall temperature	T-type thermocouple	1 K or 1.5% below 273 K
P_c_	Tank pressure	Kulite CTL-190M140BARA	7 kPa
δ	Tank wall thickness	Caliper	0.03 mm
ṁ	LN_2_ mass flow rate	Coriolis flow meter	0.3%
M	Mass of the fluid components (tube, tee, nozzle)	scale	0.1 g

### Reporting summary

Further information on research design is available in the Nature Research Reporting Summary linked to this article.

## Results

### Microgravity experiments completed

The University of Florida flight team led by Professor Jacob Chung performed parabolic flight experiments in four flights during Zero-g Corporation’s fight week^19^ of November 11–16, 2019. Table 2 lists the eight cases we performed during the parabolic flight microgravity experiment. As mentioned above, the gravity level under simulated microgravity environment in the parabolic flight is about ±0.01 g where g is earth gravity. In Table 2, P_in_ is the inlet pressure measured at the supply dewer. First, we need to stress that the mass flow rate from the spray nozzle is actually proportional to the inlet pressure as the flow is driven by the pressure difference between the inlet pressure and the pressure inside the test tank. Since the tank pressure is relatively constant that makes the mass flow rate to be totally dependent on the inlet pressure. As a result, it is the case that the higher the inlet pressure, the higher the mass flow rate. Also in Table 2, for the only pulse flow, a 50% duty cycle (DC) means the valve is open half of the time in a period. Therefore, 100% DC is a continuous flow. The pulse flow is not applicable to NVF cases.

**Table 2:** Test conditions for the 8 cases

Case	P_in_ (psig) 1 psig = 6.89 kPag	Duty Cycle (%)	Period (second)	Process
1	40	100	NA	CHV
2	70	100	NA	CHV
3	70	50	2	CHV
4	100	100	NA	CHV
5	40	NA	NA	NVF
6	70	NA	NA	NVF
7	80	NA	NA	NVF
8	90	NA	NA	NVF

### CHV thermal-fluid transport

For the CHV in the microgravity period during each parabola, the CHV procedure starts with charging a small amount of liquid by the spray nozzles onto the tank wall to chill down the tank wall, while holding the vent valve closed to allow the liquid to completely vaporize, and then opening the vent valve to relieve the pressure. This three-step procedure is repeated during the microgravity period of each of the five parabolas. In the current experiment, after 3 s, the spray is turned off and the process enters the “Hold” phase where the vent valve stays closed to allow the generated cool vapor to further chill down the tank wall. The hold-phase lasted the rest of the microgravity period that is about 14–17 s. Then the vent valve is opened to release most of the vapor during the 60-second non-microgravity transition to next microgravity period. The CHV process was repeated during each of the next four microgravity periods. In our flights, Zero-G flew five parabolas consecutively in a 5-parabola set. As a result, five successive CHV runs comprise one complete test.

**Fig. 10:**
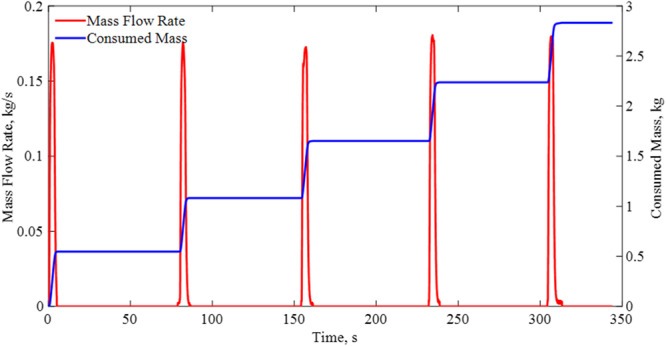
Total mass flow rate to the nozzzle during CHV under a source pressure of 100 psig and continuous flow (Case 4). The red line is the instantaneous flow rate and the blue line shows the accumulative mass consummed.

Figure 10 shows the time-dependent total mass flow rate supplied to the four nozzles by the source dewer for a typical case (Case 4, 100 psig (689 kPag) and continuous flow). Figure 10 also provides the cumulative consumed LN2 mass. The flow is in a pulse form with a duration of 3 s.

Before we present and discuss the major results of tank wall temperature history during spray quenching of the tank wall within CHV, the physics and characteristics of the tank wall heat transfer must be clearly explained first. Since the spray cooling of the tank wall is characterized by the rate of wall temperature changes, naturally we need to track the wall temperature history during chilldown. The tank wall transient temperature history is called the “chilldown curve” as explained in our previous papers^14,15^.

In the parabolic flight experiment, the test procedure included initially preparing the tank wall for a relatively uniform temperature distribution around 280 K to 300 K. Then, during the 17–20 s of microgravity period, the CHV process begins with the charge of cooling fluid to the test tank to chill down the tank wall while the vent valve is closed. Specifically, in the current CHV design, the charge of coolant is in the form of a spray that directly impinges on the tank wall to achieve the efficient quench cooling of the wall. After 3 s, the spray is stopped and the process enters the “Hold” phase where the vent calve stays closed to allow the generated cool vapor to further chill down the tank wall. The hold-phase lasted the rest of the microgravity period that is about 14–17 s. Then the vent valve is opened to release most of vapor. The CHV process was repeated during each of the next four microgravity periods. In our flights, Zero-G flew five parabolas consecutively in a 5-parabola set. As a result, five successive CHV runs comprised one complete test. We chose a typical case to illustrate the successful outcome of the CHV parabolic flight experiment.

**Fig. 11:**
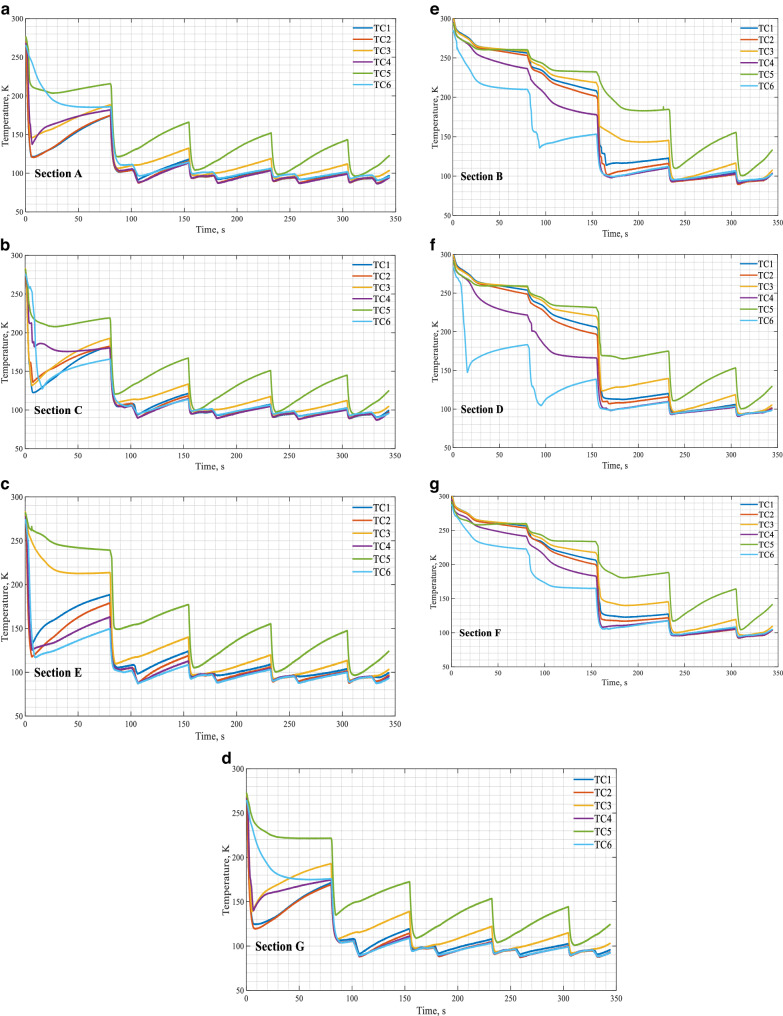
Chilldown curves during CHV under a source pressure of 100 psig and a continuous spray flow (Case 4). **a** Section A, **b** section C, **c** section E, **d** section G, **e** section B, **f** section D, **g** section F.

Again, Case 4 is used to illustrate the tank wall temperature transients. For Case 4, the 3-second spray of a continuous flow was supplied at a dewer pressure of 100 psig. A chilldown curve explained in^14,15^ for a local point on the tank wall is defined as the transient temperature history at this point recorded by the thermal couple (TC) soldered on the back of this point during the chilldown process. So, these temperature history curves or chilldown curves are the plots of the transient temperatures measured by the TCs soldered on the back of the tank wall that registered the wall back-side local surface temperature histories during chilldown. Figure 11 shows the temperature history (chilldown curve) plots for the seven sections (A, C, E, G, B, D, F) of the tank wall during CHV. In general, all six chilldown curves for each section displayed similar trends during the 3-second spray charge period, where the temperatures of all the seven walls took a steep drops during the charge period and then rose back up in the hold-period and vent period due to residual heating from the surroundings. As to the slightly different chilldown rates registered by the six TCs, it is due to the fact that the rate of heat transfer (cooling) at a given location in inversely proportional to the distance between this location and the center of the spray cone. The chilldown time is also inversely proportional to the cooling rate that results in a fact that the closer to the center of spray cone, the shorter the chilldown time. As the center of the spray cone is located between TC1 and TC2, that is why either of these two locations always has the shortest chilldown time. All seven sections were chilled down to below the target temperature of 100 K at the end of the fifth (last) CHV period that was at around the 350 s mark. Note that the lowest wall temperatures for all seven sections took place right at the end of the 3-second spray charge period during the last (5th) CHV cycle. For all the seven sections, the temperatures on the top wall surfaces measured by TC5’s were always rising fast after reaching the lowest temperatures at the end of spray charge periods that was due to the fact that they were located closest to the large thermal mass, the stainless steel flange (see Fig. 6).

Also, it needs to point out that there is a consistent and succinct difference on the characteristics of the wall chilldown curves between those in Sections A, C, E and G and those in Sections B, D, and F. For the A, C, E, and G sections, they get quenched by the spray directly, however, Sections B, D, and F do not interact with spray directly and these section are cooled down by the two neighboring sections through conduction and also by the cool vapor generated by the spray-cooled sections. For the A, C, E, and G Sections, after the wall temperatures experienced steep drops, they stayed relatively constant during the 17-second hold period. But the wall temperature showed a small drop when the vent was open before a moderate rise in temperatures over the rest of vent period. However, for the B, D, and F Sections, after the steep drop in the spray charge period, the wall temperature just followed a steadily slow rise pattern without the small drop seen in the spray wetted sections. We suggest that the small step drops in wall temperature of the wetted sections were due to the instantaneous vaporization of the liquid on the wetted wall surface when the vent was opened that lowered the tank inside pressure. The depressurization in the tank during the “Vent” stage actually provides more wall cooling that enhances the tank chilldown efficiency.

**Fig. 12:**
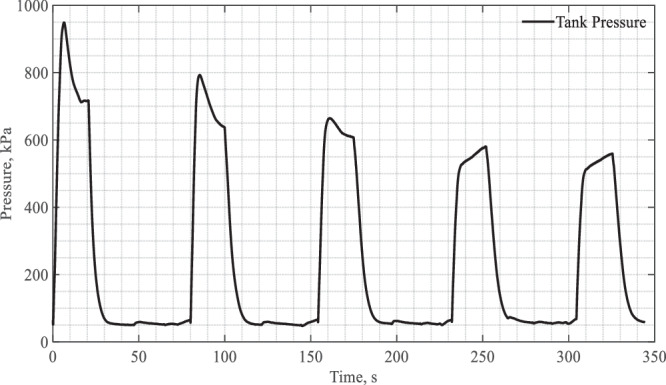
Tank pressure histogram during CHV. It was measured under a source pressure of 100 psig and continuous spray flow (Case 4).

Figure 12 depicts the pressure histogram in the test tank interior. Let us focus on the transient tank pressure history where the tank pressure showed a surge in response to each spray charging of fluid. For each of the first three CHV cycles, the pressure surge began with a sharp spike initially due to the instant flashing and vaporization of LN2 from the hot tank surface whose temperature was much higher than the Leidenfrost point. The height of the spike became lower after each charge period as the wall temperature started to decrease due to spray quenching. The initial spike disappeared after the third microgravity period as a result of the much lower wall temperatures (~110 K, see Fig. 11) which were lower than the Leidenfrost temperature. The Leidenfrost temperatures were reported to be between 145 K to 180 K for LN2 spray quenching of a circular disk^14^. When the wall surface temperature was lower than the Leidenfrost point, the spray LN2 fluid would not cause any instant flashing and vapor generation. For the last two CHV cycles, the pressure surges without the initial spikes were seen to increase slowly about 50 kPa during each of the last two CHV periods. These small pressure increases were mainly due to the local vapor generation on the upper portion of the tank wall where the temperatures were above 150 K (see TC5 readings in Fig. 11) during the last two CHV periods. The tank pressure dropped off quickly during the vent-period.

In order to measure quantitatively the CHV performance, we introduce a thermal efficiency concept that measures how much of the cooling capacity of the supplied spray cryogenic liquid actually contributes towards chilling down the tank wall during CHV. The CHV thermal efficiency as defined below represents the percent of available total quenching capacity of the liquid cryogen supplied by the source that is actually gets utilized in cooling the tank wall (target of spray cooling) from room temperature to liquid saturation temperature of LN2 corresponding to tank internal pressure. The CHV thermal efficiency, ${\eta }_{CHV}$, is therefore defined as,

1 $${\eta }_{CHV}=\frac{{Q}_{{\rm{Removed}}}}{{Q}_{Available}}\times 100 \%$$

In the above, ${Q}_{{\rm{Removed}}}$ is the total thermal energy removed from the tank wall by the spray fluid during CHV and is defined as,

2 $${Q}_{{\rm{Removed}}}=\sum\limits_{i}{M}_{i}{c}_{p,SS}({\bar{T}}_{i,initial}-{\bar{T}}_{i,final}),\,i=A,B,C,D,E,F,G,H$$

where *i* is the tank wall Section *i* and ${M}_{i}$ is the mass of the wall Section *i*. ${c}_{P,SS}$ is the stainless steel specific heat for the tank wall material. ${\bar{T}}_{i,Initial}$ and ${\bar{T}}_{i,Final}$ are the volume averaged mean initial steady state temperature of the tank wall Section *i* when the chilldown is started and the volume averaged mean final steady state temperature of this tank wall section *i* when the chilldown is completed, respectively. The end of chilldown temperature is the liquid saturation temperature corresponding to the local pressure.

${Q}_{Available}$ is the total quenching capacity provided by the spray fluid during the CHV process. It is defined as,

3 $${Q}_{Available}={M}_{coolant}{h}_{fg}$$

Where ${M}_{Coolant}$ is the total mass of spray fluid consumed and it can be estimated as,

4 $${M}_{Coolant}={\int }_{0}^{{t}_{End}}\dot{m}(t)dt$$

$\dot{m}(t)$ is the recorded time-dependent coolant mass flow rate (see Fig. 11) and ${t}_{End}$ is the end of chilldown time that corresponds to the time when the entire tank wall area has reached the end of the chilldown temperature. Therefore, ${M}_{Coolant}$ is the total mass of cryogenic coolant consumed in the entire CHV process. ${h}_{fg}$ is the latent heat of vaporization per unit mass that means ${Q}_{Available}$ is the available total quenching capacity.

To find the uncertainty in the calculated CHV thermal efficiency, ${\eta }_{CHV}$, Eqs. (1) to (4) were cast in terms of seven independent quantities. The uncertainty of the CHV efficiency was determined by applying the individual uncertainties of the seven quantities (listed in Table 3) using the root-sum-square method. The relative uncertainties for the CHV thermal efficiencies in microgravity range between 7.40% to 7.58%.

**Table 3:** Individual uncertainty of the independently measure quantities

Symbol	Quantity	Measurement Method	Uncertainty
*D*	Diameter of the target area	Rulers	1.6 × 10^−3 ^m
*ρ*	Density of test section	NIST website	1%
*c*_*p*_	Specific heat capacity of test section	NIST website	5%
*ΔT*	Temperature difference between the initial and the end of the test	T-type thermocouple	1.5%
*τ*	Thickness of test section	Calipers	3 × 10^−5 ^m
*h*_*fg*_	Latent heat of vaporization	NIST website	5%
${\dot{m}}_{l}$	Mass flow rate of liquid nitrogen	Coriolis flow meter	0.3%

Table 4 lists the calculated chilldown efficiencies using Eqs. (1)–(4) for all four CHV cases performed in microgravity. For each case, the total amount of spray mass used is also given in Table 4. With the four cases investigated, the efficiencies range between 20.40–39.49%. In general, the spray chilldown (cooling) efficiencies using cryogenic fluids are significantly higher than those using water or FC 72 that normally carry efficiencies only from 5% to up to about 10%^21,22^. On the total cryogen mass consumed, it is inversely proportional to the efficiency as expected. The minimum consumed mass is 0.8820 kg, that corresponds to the maximum efficiency at 39.49% for continuous spray flow from 40 psig supply dewer pressure. In actual space applications, the total amount of cryogen consumption is of highest concern as that affects the total payload weight at the rocket lift-off.

**Table 4:** CHV tank chilldown efficiency in microgravity

Case CHV, 3-s CHARGE, 17-s HOLD	Total Spray Mass Consumed, M_coolant_, kg	Efficiency, %
1. Continuous flow, 40 PSIG (275.6 kPag)	0.8820	39.49
2. Continuous flow, 70 PSIG (482.3 kPag)	2.0464	24.80
3. Pulse flow, 2 s/50%, 70 PSIG (275.6 kPag)	1.9431	26.31
4. Continuous flow, 100 PSIG (689 kPag)	2.8314	20.40

Based on the CHV data obtained in microgravity and terrestrial gravity, the effects of reduced gravity are assessed in terms of the total LN2 mass consumed and chilldown efficiency listed in Table 5. It is clear that gravity enhances the CHV chilldown efficiency that reflects in less LN2 consumed in terrestrial gravity. As a matter of fact, the thermal efficiency was increased by 6.9% and the LN2 mass consumption was reduced by 20.4% in terrestrial gravity. To explain the above, we offer the following. The higher thermal efficiencies found in terrestrial gravity are thought to be caused primarily by the two heat transfer enhancement mechanisms. Natural convection, that is driven by gravity, is the first enhancement mechanism. During the film boiling period of wall chilldown, the tank wall surface is covered by a vapor film where natural convection would set in due to the hot wall. Therefore, gravity-driven, buoyancy-sustained natural convection is responsible for the heat transfer enhancement during film boiling period. While draining and thinning of the liquid film by gravity during the transition and nucleate boiling periods would be the second enhancement mechanism as the thinner the liquid film, the higher the heat transfer. The current authors also reported tank chilldown results^15^ using continuous spray cooling throughout the entire tank chilldown process instead of CHV. Where it was also found that gravity enhanced tank chilldown efficiency and reduced cryogen consumption.

**Table 5:** Comparison of terrestrial and microgravity CHV performances

Case CHV, 3-s CHARGE, 17-s HOLD	Total Spray Mass Consumed, M_coolant_, kg	Efficiency, %,
1. Continuous flow, 100 PSIG (689 kPag) Earth gravity	2.35	21.80
2. Continuous flow, 100 PSIG (689 kPag) Microgravity	2.83	20.40

Let us first compare the efficiencies for Cases 2, and 3 for the flow pulsing effects as both have the same inlet pressures of 70 psig. As can be seen from Table 4 that for Case 3 with a pulse flow of 50% DC and 2 s period, its efficiency is 26.31% that is 6.1% higher than the continuous flow (Case 2) at 24.80%. While on the LN2 mass consumption comparison, the pulse flow case used 1.9431 kg of LN2 and continuous flow case used 2.0463 kg that is 5.3% more than that of pulse flow case. Therefore, it is quite consistent from both efficiency and consumed mass comparisons that pulse flow enhanced CHV performance. Pulse flows have been shown to enhanced cryogenic chilldown efficiencies for both pipe chilldown and tank chilldown in both microgravity and terrestrial gravity in our previous research^5,7–12^.

As mentioned above, the mass flow rate is actually proportional to the inlet pressure that results in a condition that the higher the inlet pressure, the higher the mass flow rate. Therefore, we are also examining the effect of various nozzle mass flow rates. For continuous flow Cases 1, 2, and 4 with inlet pressures of 40 psig, 70 psig and 100 psig, the measured efficiencies are 39.49%, 24.80%, and 20.40%, respectively. It is clear that the chilldown efficiency is inversely proportional to the mass flow rate and inlet pressure. Similar inlet pressure effects have been found for both pipe chilldown and tank chilldown in both microgravity and terrestrial gravity in our previous research^5,7–12^.

Since tank chilldown is a transient process, it is instructive to evaluate how does the chilldown efficiency vary with time. We use Case 4 to examine the time-dependence of CHV tank chilldown. Table 6 lists the HV chilldown efficiencies in terms of total number of microgravity periods involved. For example, 1 + 2 means that the CHV chilldown has gone through microgravity period 1 (parabola 1) and microgravity period 2 (parabola 2). 1 + 2 + 3 + 4 + 5 means the CHV chilldown has gone through all five microgravity periods (all 5 parabolas). AS we can see clearly that the CHV chilldown efficiency decreases with increasing CHV processes. The main reason for this efficiency deterioration is that the the difference between the tank surface temperature and cooling fluid temperature, that is the heat transfer driving force, decreases as time goes on due to the tank surface gets chilled down.

**Table 6:** CHV chilldown efficiency as a function of total number of parabolas involved

Number of Parabolas involved	Consumed Mass, kg	Efficiency, %
1	0.5459	48.83
1 + 2	1.0827	38.76
1 + 2 + 3	1.6510	34.08
1 + 2 + 3 + 4	2.2376	26.40
1 + 2 + 3 + 4 + 5	2.8314	20.40

In theory, there are different ways you can chill down a tank wall. The current authors’ group has tried two different methods. The first method, called “pure spray cooling tank chilldown” is to use spray cooling continuously until the wall is chilled down to liquid temperature. The second method is the current “CHV” method. The current authors have reported the pure spray cooling results in^15^. Table 7 provides a comparison of chilldown efficiencies between CHV and pure spray cooling. Based on Table 7, it is apparent that tank chilldown by CHV is much more efficient than by pure spray cooling for lower source pressure (i.e. lower mass flow rate) at 40 psig. As the source pressure was increased to 70 psig and then to 100 psig, the two methods are fairly comparable to each other.

**Table 7:** Comparison between CHV and pure spray cooling

Case	Total LN2 Mass Consumed, M_coolant_, kg CHV	Total LN2 Mass Consumed, M_coolant_, kg Spray Cooling	Efficiency, % CHV	Efficiency, % Spray Cooling
Continuous flow 40 PSIG	0.8820	1.1858	39.49	28.22
Continuous flow 70 PSIG	2.0464	1.5332	24.80	24.49
Continuous flow 100 PSIG	2.8314	2.5551	20.40	21.08

### NVF thermal-fluid transport

For the NVF experiment performed during the parabolic flights, the test tank was fully chilled down to begin the experiment. The filling of the tank by the spray of four nozzles was initiated when the microgravity period began and the vent valve was closed unless the tank pressure went over the 105 psig (723.5 kPag) safety threshold for the vent to open. The filling was then stopped when the microgravity period ended. The NVF process was repeated during each of the next four microgravity periods.

**Fig. 13:**
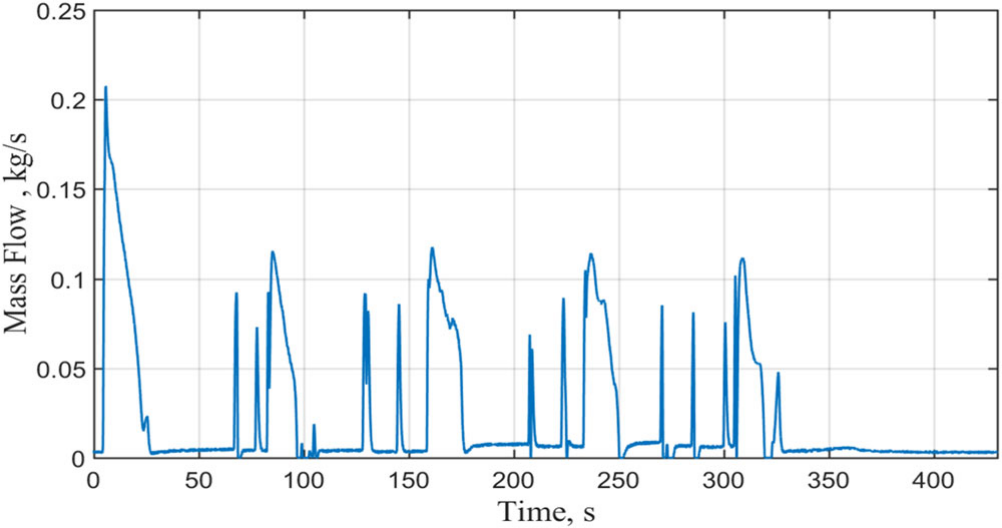
Mass flow rate history during the NVF. It was measured under 90 psig source pressure with a continuous flow (Case 8).

**Fig. 14:**
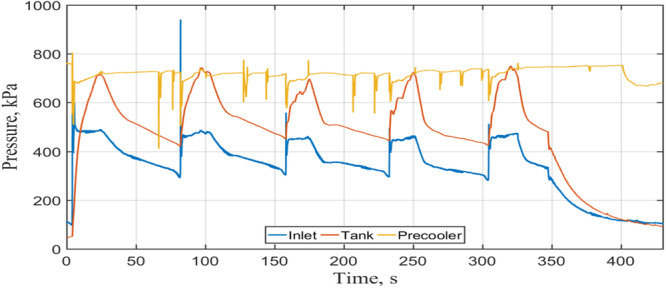
Tank pressure history during the NVF. It was measured under 90 psig source pressure with a continuous flow (Case 8).

During our flights, Zero-G flew five parabolas consecutively in a 5-parabola set. As a result, five successive NVF runs comprised one complete NVF test. Next, typical NVF results are presented for Case 8 listed in Table 2. Case 2 was performed with 90 psig dewer source pressure and continuous flow supply. Figure 13 shows the mass flow rate history during the fill period where five filling periods are seen. A mass flow rate spike was seen during each fill period. The first fill period has the highest peak value as the back pressure in the test tank was the lowest during the first fill period. For the rest four filling periods, the tank pressure was built up to much higher values (see Fig. 14).

**Fig. 15:**
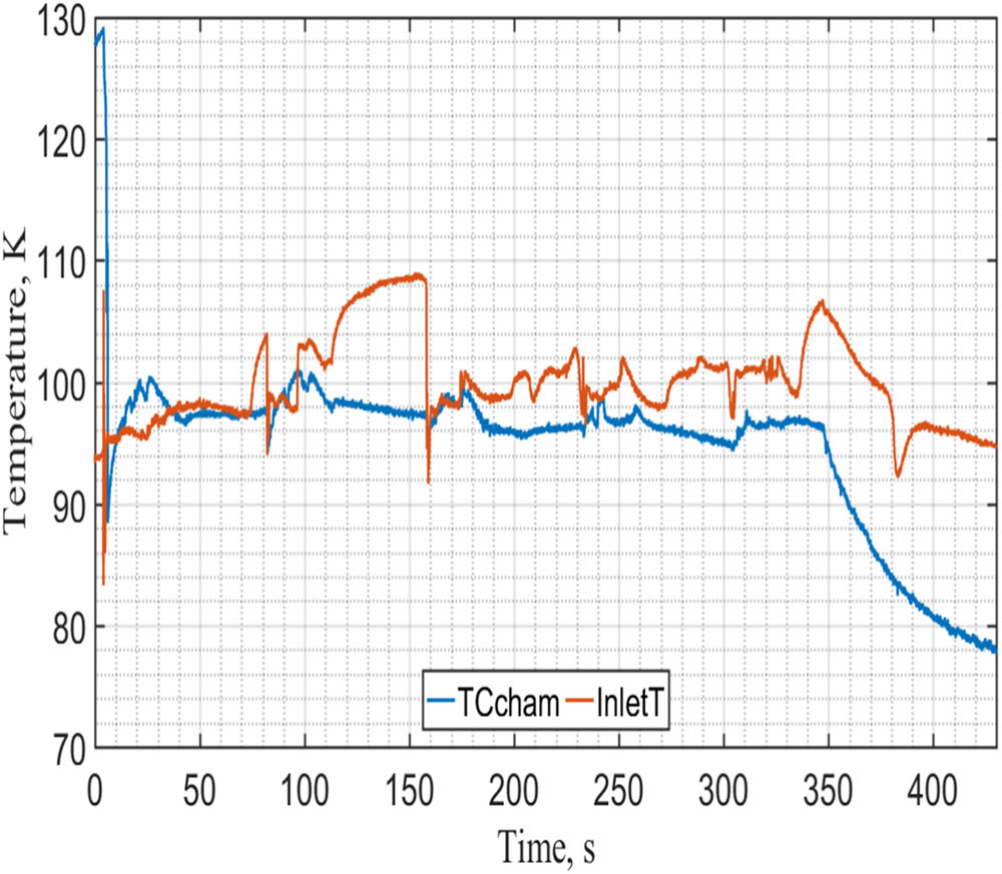
Tank temperature history during NVF. It was measured under 90 psig of source pressure with continuous flow (Case 8).

**Fig. 16:**
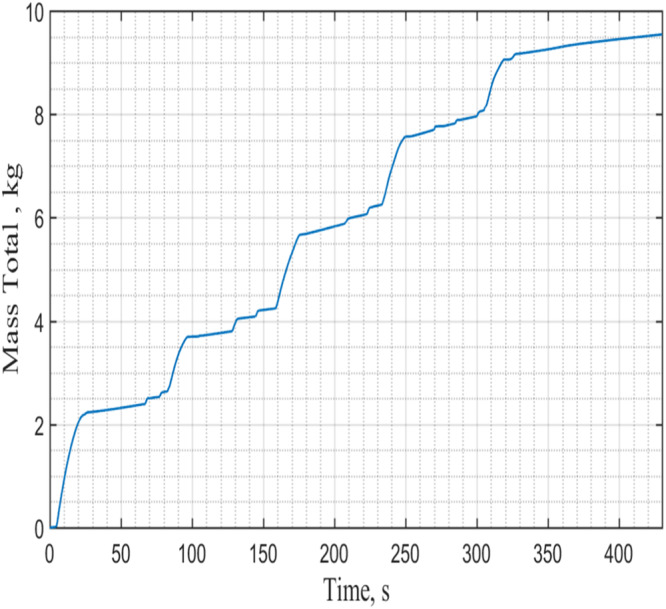
Total LN2 mass supplied to the tank during NVF. It was measured under 90 psig source pressure with continuous flow (Case 8).

Figure 14 provides the measured tank pressure history during NVF. As can be seen that, the test tank inside pressure would reach the vent pressure of 105 psig (723.5 kPag) at the end of each microgravity period when the vent of vapor would take place. Figure 15 displays the corresponding tank temperature history. It is clear that the tank inside temperature is the corresponding saturation temperature of the tank pressure inside the tank, it is in the vapor-liquid mixture saturated state. Next, Fig. 16 gives the total mass supplied to the tank as a function of time. For this case, the total mass supplied to the tank is 9.5 kg at the end of NVF experiment. As the tank total capacity is 6 kg, according to Fig. 16, the tank would have been completely filled up in about three microgravity periods if venting were not required. The venting of vapor was needed in our experiment mainly due to the residual heating during the 60-second high-g periods between the microgravity periods. In other words, if we were given three consecutive 17-second microgravity periods, the 6-kg tank would have been completely filled out without any venting. We can therefore conclude that the NVF experiment in microgravity was basically successful.

### General conclusion

The feasibilities of both CHV and NVF have been demonstrated in simulated microgravity. For CHV tests, we found that all seven sections were chilled down to below the target temperature of 100 K at the end of the fifth (last) CHV period. Of the particular advantage from the CHV procedure is that the depressurization in the tank during the “Vent” stage actually provides more tank wall evaporative cooling that enhances the tank chilldown efficiency. With the four cases investigated, the CHV thermal efficiencies ranged between 20.40–39.49%. It is clearly indicated by the results that gravity enhances the chilldown efficiency that reflects in more LN2 consumed in microgravity. As a matter of fact, the thermal efficiency was decreased by 6.9% and the LN2 mass consumption was increased by 20.4% in microgravity. If the spray charge flow is pulsed, from both CHV thermal efficiency and consumed mass comparisons, we found that pulse flow enhanced CHV performance with a 6.1% higher thermal efficiency and 5.3% lower LN2 mass consumption. The results also suggest that the CHV chilldown efficiency is inversely proportional to the spray mass flow rate and inlet pressure. As a result, operating the CHV process at lower mass flow rates could produce higher efficiencies that means more cryogen savings. In reality, a lower spray mass flow rate would require a longer chilldown time that would result in more residual heating. Therefore, if we were to include the residual heating, there should exist an optimal spray mass flow rate. Also, it is apparent that tank chilldown by CHV is much more efficient than by pure spray cooling for lower source pressure (i.e. lower mass flow rate) at 40 psig. As the source pressure was increased to 70 psig and then to 100 psig, the two methods are fairly comparable to each other. The feasibility of tank filling by NVF was successfully demonstrated in microgravity.

Our main objective was to introduce highly energy efficient, thermal-fluid management breakthrough concepts to conserve and minimize the cryogen consumption during propellant storage tank chilldown in space, it is believed that we have accomplished this objective. The results from microgravity experiments show that through the combination of spray cooling, hold, vent, and flow pulsing, the feasibility of a highly efficient propellant storage tank chilldown technology has been established.

### Supplementary information

Reporting Summary

## Data Availability

The authors declare that the data supporting the findings of this study are available within the paper.

## References

[1] Mars Architecture Steering Group. Human exploration of Mars Design Reference Architecture 5.0. (ed. Drake, B. G.). Report No. NASA/SP-2009-566 (National Aeronautics And Space Administration) (2009).

[2] Meyer ML (2013). Mastering cryogenic propellants. J. Aerosp. Eng..

[3] Motil, S. M., Meyer, M. L., Tucker, S. P. Cryogenic fluid management technologies for advanced green propulsion systems. *AIAA 45th Aerospace Sciences Meeting and Exhibit**;* NASA/TM-2007-214810; Reno, NV, January 8th, 2007.

[4] NASA. NASA Technology Roadmaps, TA 2: In-Space Propulsion Technologies (National Aeronautics and Space Administration) (2015).

[5] Shaeffer R, Hu H, Chung JN (2013). An experimental study on liquid nitrogen pipe chilldown and heat transfer with pulse flows. Int. J. Heat. Mass Transf..

[6] Doherty, M. P. et al. Cryogenic fluid management technology for Moon and Mars missions. *NASA Report NASA/TM—2010-216070* (2010).

[7] Darr SR (2016). The effect of reduced gravity on cryogenic nitrogen boiling and pipe chilldown. Nat. Partn. J. (NPJ) : Microgravity.

[8] Chung JN (2021). An advance in transfer line chilldown heat transfer of cryogenic propellants in microgravity using microfilm coating for enabling deep space exploration. Nat. Partn. J. (NPJ): Microgravity.

[9] Chung JN, Darr SR, Dong J, Wang H (2019). Enhancement of convective quenching heat transfer by coated tubes and intermittent cryogenic pulse flows. Int. J. Heat. Mass Transf..

[10] Chung JN, Darr SR, Dong J, Wang H, Hartwig JW (2020). Heat transfer enhancement in cryogenic quenching process. Int. J. Therm. Sci..

[11] Hartwig J (2022). Nitrogen flow boiling and chilldown experiments in microgravity using pulse flow and low-thermally conductive coatings. Nat. Partn. J. (NPJ): Microgravity.

[12] Hartwig J (2023). The effect of gravity on cryogenic transfer line chilldown performance using pulse flow and low thermally conductive coatings. Int. J. Heat. Mass Transf..

[13] Keefer KA, Hartwig JW (2016). “Development and validation of an analytical charge-hold-vent model for cryogenic tank chilldown”. Int. J. Heat. Mass Transf..

[14] Chung, J. N., Dong, J., Wang, H., Darr, S. R., Hartwig, J. W. Cryogenic spray quenching of a simulated propellant storage tank wall with heat transfer enhancement by a thin-film coating and flow pulsing in microgravity, *Nat. Partner J. (NPJ)*: Microgravity, 8 (2022) 7. 10.1038/s41526-022-00192-w PMC8975813 35365683

[15] Chung JN, Dong Jun, Wang Hao, Huang BH (2022). Effects of microgravity on cryogenic spray quenching of a simulated propellant storage tank with enhancement by surface coating and flow pulsing. Int. Comm. Heat. Mass Transf..

[16] Chato, D. J. & Sanabria, R. Review and test of chilldown methods for space-based cryogenic tanks. *27th**Joint Propulsion Conference*, Sacramento, CA, June 24–27, (1991).

[17] Honkonen SC, Pietrzk JR, Schuster JR (1992). Analysis of pulsed injection for microgravity receiver tank chilldown. Adv. Cryog. Eng..

[18] Konishi C, Mudawar I (2015). Review of flow boiling and critical heat flux in microgravity. Int. J. Heat. Mass Transf..

[19] Zero-g Corporation, https://www.incredible-adventures.com/zero-g-how.html.

[20] DuPont^TM^ Teflon FEP, Fluoroplastic Film (2013), WWW.teflon.com/Industrial.

[21] Liang G, Mudawar I (2017). Review of spray cooling – Part 1: single-phase and nucleate boiling regimes, and critical heat flux. Int. J. Heat. Mass Trans..

[22] Liang G, Mudawar I (2017). Review of spray cooling – Part 2: high temperature boiling regimes and quenching applications. Int. J. heat. Mass Trans..

